# Features of self-attitude and self-esteem of freelancers

**DOI:** 10.1192/j.eurpsy.2023.2068

**Published:** 2023-07-19

**Authors:** V. Umlauft

**Affiliations:** Social psychology, Lomonosov Moscow State University, Vienna, Austria

## Abstract

**Introduction:**

Freelancing can be analyzed through psychological prism of escapism, through the desire not to be involved in the systems of inflexible social ties that are accepted in group (career, family, classical education, etc.)

**Objectives:**

Freelancers have a non-standard self-attitude and self-description, which indicates their “isolation” from the real social world by these people.

**Methods:**

Quantitive (**Spyrman’s criteria** and corellations) and qualitive analisys ( semi-structured interviews). N = 300, residents of ussia and Austria.

**Results:**

The hypothesis was rejected that freelancers do not have career and personal orientations, in contrast to individuals who are not prone to this type of career choice. The absence of a connection between goal setting and downshifting was revealed based on the analysis of the author’s questionnaire for goal setting.

The hypothesis was empirically confirmed that freelancers have more pronounced indicators such as global self-attitude, self-esteem, self-sympathy, expected attitude from others, self-confidence, self-acceptance in the affective component of the self-concept, in contrast to people choosing the traditional career path.

**Conclusions:**

Freelancers have a less pronounced indicator of self-accusation, in contrast to people committed to careerism.

**Disclosure of Interest:**

None Declared

